# *De novo* sequencing allows genome-wide identification of genes involved in galactomannan synthesis in locust bean (*Ceratonia siliqua*)

**DOI:** 10.1093/dnares/dsae033

**Published:** 2024-12-02

**Authors:** Mitsuaki Akutsu, Akihisa Shinozawa, Tomoaki Nishiyama, Yoichi Sakata, Yuji Hiwatashi

**Affiliations:** Graduate School of Food, Agricultural and Environmental Sciences, Miyagi University, Sendai 982-0215, Japan; Aoba Kasei Co., Ltd, Sendai 981-3137, Japan; Department of Bioscience, Tokyo University of Agriculture, Tokyo 156-8502, Japan; Research Center for Experimental Modeling of Human Disease, Kanazawa University, Kanazawa, Ishikawa 920-0934, Japan; School of Science, Academic Assembly, University of Toyama, Toyama, 930-8555, Japan; Department of Bioscience, Tokyo University of Agriculture, Tokyo 156-8502, Japan; Graduate School of Food, Agricultural and Environmental Sciences, Miyagi University, Sendai 982-0215, Japan

**Keywords:** *Ceratonia siliqua*, genome, transcriptome, galactomannan

## Abstract

Locust bean (*Ceratonia siliqua*) accumulates the galactomannan (GM) locust bean gum (LBG) in its seeds. LBG is a major industrial raw material used as a food thickener and gelling agent, whose unique properties mean that it cannot be readily replaced by other GMs. Whereas much is known about GM accumulation and the genes associated with GM biosynthesis in legumes, the genes involved in GM biosynthesis in *C. siliqua* are largely unknown. Here, we present a genome-wide list of genes predicted to be associated with the GM biosynthesis pathway in *C. siliqua.* We confirmed high GM accumulation in endosperm using a newly established GM quantification method involving LC-MS/MS. Through *de novo* draft genome assembly, we comprehensively identified genes predicted to be related to the GM biosynthesis pathway in *C. siliqua* by identifying orthologous groups. In particular, we identified all genes predicted to encode mannan synthase (ManS) and galactomannan galactosyltransferase (GMGT), enzymes functioning in the final step of GM biosynthesis, from the *C. siliqua* draft genome. *ManS* and the *GMGT* paralogs were predominantly expressed in endosperm. The genome and transcriptome produced in this study should facilitate research examining why *C. siliqua* produces LBG, unlike other legumes.

## 1. Introduction

**Fig. 1.**
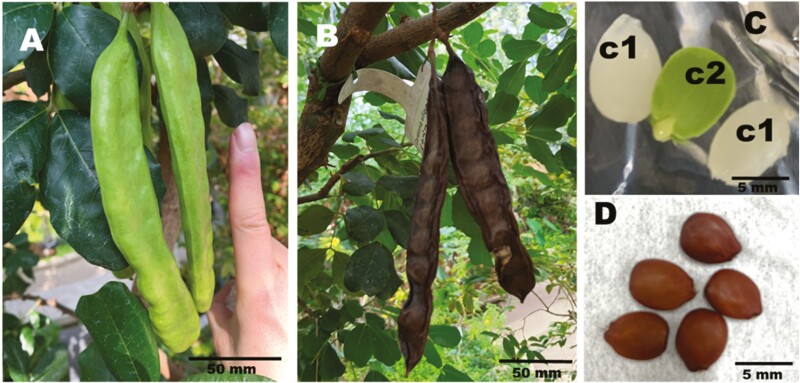
The fruits and seeds of locust bean. (A) Immature fruits 4 mo after flowering (photographed at Sakuya Konohana Kan). (B) Mature fruits 10 mo after flowering (photographed at Sakuya Konohana Kan). (C) Endosperm (c1) and cotyledons (c2) of immature seeds 4 mo after flowering. (D) Mature seeds.

*Ceratonia siliqua* L. (commonly known as locust bean) is a woody plant primarily found along the Mediterranean coast. *C. siliqua*, a member of the Fabaceae subfamily Caesalpinioideae, can reach over 10 m in height, thrives in warm climates, and is highly resilient to dry conditions. This species is typically dioecious, with separate male and female trees. Its fruit consists of elongated, angular pods reminiscent of locusts, leading to the name locust bean (Fig. 1A and B). It takes ~8 yr from planting to harvest locust bean fruit and ~1 yr for the fruit to ripen.^1^ The fruit contains sugars, making it sweet, and can be consumed fresh or dried as a food ingredient as well as a food.

The endosperm that forms during locust bean seed development contains galactomannan (GM) (Fig. 1C and D). GM is a valuable material used to produce a food-grade thickener called locust bean gum (LBG) that is widely utilized in the food industry.^2^ GM exists as a storage polysaccharide in the endosperm of many legume species, serving as an excellent source of carbohydrate energy for developing seeds and enhancing drought resistance during pre- and post-germination.^3^ The commercial utility of various GMs from legumes depends on their chemical properties. For example, the GM from *C. siliqua* (i.e. LBG) seeds contains one galactose bound to the C6 position for every 4 mannoses in the mannan main chain.^4^ The GM in *Cyamopsis tetragonoloba* (guar bean) seeds is also utilized as a food additive, called guar gum, but has a different structure, in which one galactose is linked to 2 mannoses.^4^ Because of these distinct chemical properties, *C. siliqua* GM (LBG) is commonly used as both a thickening agent and a gelling agent, whereas *C. tetragonoloba* GM guar gum is primarily employed as a thickener. Therefore, LBG is indispensable for adjusting the texture of gels and cannot be readily replaced with guar gum or other GMs.

One clear difference among GMs in legumes is their sugar composition. However, the mechanism that regulates the sugar composition of GMs and their accumulation in the endosperm of leguminous seeds is largely unknown. Endosperm extracts from *Trigonella foenum-graecum* (fenugreek) and *C. tetragonoloba* showed *in vitro* GM synthesis activity,^5^ although the enzymes for GM biosynthesis were not identified. In *C. siliqua*, the possible biosynthesis of GM was detected in cultured protoplasts derived from cotyledons, suggesting that GM is biosynthesized during cell wall regeneration.^6,7^ This suggests that GM biosynthesis may take place during periods other than endosperm development, although it is unclear whether other types of cells exhibit GM biosynthesis.

GM biosynthesis likely varies among leguminous species; in particular, galactose is bound to the mannan backbone at particular ratios in a species-dependent manner,^8^ with varying ratios of galactose to mannose.^9^ For example, the galactose-to-mannose ratios in GM isolated from *C. tetragonoloba* and *C. siliqua* are 1:2 and 1:4, respectively.^4,10^ These differences are thought to be due to the differing activities of 2 enzymes involved in the GM biosynthesis pathway: mannan synthase (ManS), which is responsible for synthesizing mannan, a major backbone structure consisting of mannose; and galactomannan galactosyltransferase (GMGT), which transfers galactose to the mannan backbone. In *C. tetragonoloba*, ManS utilizes GDP-mannose, a precursor of the major mannan backbone, as a substrate. In *T. foenum-graecum*, GMGT catalyses the addition of galactose from UDP-galactose to the mannan backbone.^11^ The specific activity of GMGT is thought to function in the substitution of the galactosyl side chain during GM biosynthesis.^9^ Transcriptome analysis of *C. tetragonoloba* and *T. foenum-graecum* revealed a set of genes encoding the enzymes involved in GM biosynthesis.^12–14^ These findings provide valuable insights into the regulation of GM biosynthesis in leguminous plants.

To date, the genes encoding the enzymes involved in the GM biosynthesis pathway of *C. silique* remain unclear due to limited information about its genes and proteins, despite reports of expressed sequence tag (EST)^15^ and restriction site–associated DNA sequencing (RADSeq) analyses.^16^ Recently, genome information for *C. siliqua* was published.^17^ However, the genome was assembled using short-read data, and comprehensive gene annotation is still lacking. Consequently, the information is currently insufficient for genome-wide identification of orthologous genes, including those involved in GM biosynthesis. Identifying the genes encoding the enzymes involved in GM biosynthesis (e.g. ManS and GMGT) is crucial for elucidating GM formation in *C. siliqua*, for transcriptional profiling of developing endosperm and other tissues to explore the regulation of gene expression during GM biosynthesis, and for facilitating genome editing in *C. siliqua* for purposes such as enhancing GM biosynthesis.

In this study, we conducted genome-wide identification of orthologs of genes encoding enzymes for GM biosynthesis in other species. We quantified GM levels in various tissues, including endosperm, using a newly established method involving LC-MS/MS. We then produced a draft genome assembly by combining long- and short-read sequencing technologies, facilitating comprehensive identification of orthologous genes across the land plants. Finally, we performed transcriptome profiling of various tissues to explore the regulation of gene expression within the GM biosynthesis pathway. We identified one ManS gene and one GMGT gene that are predominantly expressed in endosperm and are likely involved in GM biosynthesis.

## 2. Materials and Methods

### 2.1. Plant materials

Mature leaves and stems were collected from a *C. siliqua* tree growing in a greenhouse in the Koishikawa Botanical Garden, Graduate School of Science, University of Tokyo (Tokyo, Japan). Immature pods were collected from a *C. siliqua* tree growing in a greenhouse in the Sakuya Konohana Kan Conservatory (Osaka, Japan). Although the ages of both trees are unknown, they are female trees that were planted before 1990. Callus formation was induced from leaf sections grown on MS agar medium containing 5.0 µM 1-naphthaleneacetic acid and 4.4 µM 6-benzylaminopurine. The explants were incubated at 25 °C in the dark for 8 wk, and the calli were used after 6 wk of subculture.

For flow cytometry, *Brachypodium distachyon*, rice (*Oryza sativa*), and Arabidopsis (*Arabidopsis thaliana*) plants were grown at 25 °C under long-day conditions (16-h-light/8-h-dark cycle) in an incubator, and tomato (*Solanum lycopersicum*) and soybean (*Glycine max*) plants were grown in a greenhouse.

### 2.2. Flow cytometry

Mature leaves were immersed in extraction solution (10 mM Tris-HCl, 2 mM MgCl_2_, 0.1 % [v/v] Triton X-100, 0.01 % [w/v] RNase A) and cut up on ice using a razor blade. After a 10-min incubation, the extraction solution containing nuclei was collected through a cell strainer with a mesh size of 20 µm. Propidium iodide was added to the nucleus extract, and after 10 min of incubation, flow cytometry was performed using a CytoFLEX S flow cytometer (Beckman Coulter, Fullerton, CA, USA). The genome sizes of each species except *C. siliqua* were based on information in NCBI’s taxonomy database (https://www.ncbi.nlm.nih.gov/data-hub/taxonomy/), and a calibration curve was created by measuring the fluorescence intensity of each species to estimate the genome size of *C. siliqua*.

### 2.3. Nucleic acid extraction

To extract genomic DNA, mature *C. siliqua* leaves were frozen in liquid nitrogen and ground into a powder using a mortar and pestle. Genomic DNA was extracted and purified using a NucleoSpin Plant II Midi Kit (Macherey-Nagel, Duren, Germany) according to the manufacturer’s protocol. For RNA extraction, stems with the epidermis removed, endosperm, and calli were frozen in liquid nitrogen and ground into a powder using a mortar and pestle. Total RNA was extracted from the samples using PureLink Plant RNA Reagent (Thermo Fisher Scientific, Waltham, MA, USA) and further purified using an RNeasy Plant Mini kit (Qiagen, Limberg, Netherlands) following the manufacturer’s protocol with some modifications. Due to the high amount of impurities, after PureLink liquid treatment, the supernatant was subjected to lithium chloride precipitation instead of ethanol precipitation. The precipitate was suspended in buffer RLT included in the RNeasy Plant Mini kit and processed using the kit to obtain total RNA.

### 2.4. Library construction

The genomic DNA (1 μg) was fragmented using a Covaris LE220 DNA Sonication system (Covaris, Woburn, MA, USA) and purified using a QIAquick PCR Purification kit (Qiagen) to obtain genomic DNA fragments with an average length of 511 bp. The PCR-free protocol was used with a KAPA Hyper Prep Kit for Illumina (Kapa Biosystems, Wilmington, MA, USA) to prepare short-read sequencing libraries. To construct long-read sequencing libraries, a SMRTbell Express Template Prep kit 2.0 and a SMRTbell Enzyme Clean Up kit 2.0Pv2 (Pacific Biosciences, Menlo Park, CA, USA) were used to prepare libraries from genomic DNA, which were then purified using AMPure PB (Pacific Biosciences) and size-selected using the BluePippin system (Sage Science, Inc., Beverly, MA, USA) to obtain 15- to 20-kb libraries. For transcriptome analysis, a TruSeq Stranded mRNA Sample Preparation kit (Illumina, San Diego, CA, USA) was used to purify mRNA from total RNA (1,000 ng) derived from leaves, stems, endosperm, and calli and to construct sequencing libraries.

### 2.5. Analysis pipeline for NGS sequencing data

Briefly, the NGS sequencing data were analysed using the pipeline shown in Supplementary Fig. S1; details are provided in the following sections.

### 2.6. Genome sequencing and assembly

The genome size of *C. siliqua* was previously proposed to be ~600 Mbp,^18^ which was confirmed by flow cytometry, and a depth of coverage of 100 × was set based on this size. Short-read sequencing was performed using a NextSeq500 system (Illumina) with NextSeq High 150 × 2 bp (Illumina) sequencing reagents, producing 135.7 Gb of raw data. Data analysis was performed on a PC (Ryzen9 5950X, RAM128GB, Ubuntu20.4). The quality of the sequencing data was confirmed using FastQC ver.0.11.9.^19^ The raw data were processed using FASTX-Toolkit ver. 0.0.14^20^ to remove adapter sequences, trim the 5 bases at the 3ʹ ends, and filter reads containing signals with a QV value of < 30 by 20% or more. The processed read data were analysed using KmerGenie^21^ to predict the optimal *k*-mer, and GenomeScope ver.2.0^22^ was employed to estimate the genome size.

Assembly was performed using ABySS ver.2.1.5,^23^ Velvet ver.1.2.10,^24^ and SOAPdenovo2^25^ to generate contigs. The contigs were evaluated using assembly-stats ver.17.02.^26^ Long-read sequencing was performed using a Sequel IIe system (Pacific Biosciences) with a Sequel II SMRT Cell 8M Tray (Pacific Biosciences), Sequel II Sequencing 2.0 kit (Pacific Biosciences), Sequel II Binding kit 2.0, and Internal Control 1.0 (Pacific Biosciences) sequencing reagents, and single-molecule real-time sequencing was performed for 30 h, resulting in 29.1 Gb of raw data. The resulting read data were converted to FASTQ format using SMRT Link ver.10.1 (Pacific Biosciences), and reads that passed full pass 3 times or more and had a QV ≥ 20 were extracted. Assembly was performed using RAVEN ver.1.7.0,^27^ Fly ever.2.9,^28^ Canu ver. 2.2,^29^ MECAT2 ver. 20190314,^30^ Wtdbg ver. 2.5,^31^ Apollo,^32^ Rust-mdbg ver. 1.0.1,^33^ JumboDB ver. 1.0.1,^34^ IPA,^35^ and Hifiasm ver. 0.16.0.^36^ The resulting contigs were evaluated using assembly-stats ver. 17.02. The assembly with the highest N50 value was chosen as the pre-draft genome, and its completeness was evaluated using 4 core gene sets (Eukaryota_odb10, Embryophyta_odb10, Eudicots_odb10, and Fabales_odb10) with BUSCO v5.4.6.^37^

The mitochondrial and plastid genomes were *de novo* synthesized from short-read data using GetOrganelle ver. 1.7.6.1.^38^ The pre-draft genome, mitochondrial genome, and plastid genome were mapped to long-read data using minimap2 ver. 2.24,^39^ and only reads that mapped to the pre-draft genome were collected using SAMtools ver. 1.13.^40^ Of the 2,079,729 reads, 1,931,132 reads were collected and assembled using Hifiasm, and the assembly graph was drawn using Bandage ver. 0.9.0^41^ to confirm the entire structure. The assembly data were corrected using Inspector ver. 1.2^42^ with long-read data and further polished using ntEdit ver. 1.3.5^43^ with short-read data. The resulting data were used as the final draft genome. The telomere regions of each contig were analysed using TelomereSearch^44^ with a threshold of 0.4 (i.e. more than 40% of the 200 residues at the end of each contig were identified as telomeric).

### 2.7. RNA sequencing and assembly

Transcriptome sequencing (RNA-seq) was performed using the NovaSeq 6000 platform (Illumina) in 150-bp paired-end sequencing mode, resulting in raw data of several gigabases per sample. The BCL (binary base call) files were converted into FASTQ format using the Illumina package bcl2fastq. Data quality was confirmed using FastQC ver. 0.11.9. Raw data were processed using FASTX-Toolkit ver. 0.0.14, through a process that involved removing adapter sequences, trimming 12 bp from the 5ʹ ends, discarding reads that were < 80 bp, and filtering out reads containing signals with QV values < 30% in the quality filter. Preprocessed read data were assembled using Trinity ver.2.14.0.^45^ The assembly was evaluated using the utility TrinityStats.pl included in Trinity, an expression matrix was created using ‘abundance_estimates_to_matrix.pl’, and Ex90N50^46^ was obtained using ‘contig_ExN50_statistic.pl’. Completeness in each of the 4 core gene sets (Eukaryota_odb10, Embryophyta_odb10, Eudicots_odb10, and Fabales_odb10) was evaluated using the ‘-m tran’ option of BUSCO ver. 5.4.6.

### 2.8. Analysis of repeat elements

After construction of species-specific models using RepeatModeler ver. 2.0.3^47^ with LTRStruct, repeat sequences were annotated using RepeatMasker ver. 4.1.1^48^ with Dfam ver. 3.7^49^ in hmmer mode.

### 2.9. Gene prediction

The draft genome was subjected to gene prediction using AUGUSTUS ver. 3.5.0.^50^ For gene prediction, the Fabales_odb10 core gene set was used as training data with the ‘--augustus --long’ option in BUSCO. Additionally, 3 hint files were combined to enhance gene prediction with AUGUSTUS. Each hint file was created following the methodologies detailed on the official website (http://bioinf.uni-greifswald.de/bioinf/wiki/pmwiki.php?n=Augustus.Augustus). The first hint file was based on cDNA assembly data generated by Trinity to provide transcriptomic insights. The second hint file was derived from the results of RepeatMasker, providing essential information on repeat sequences. The third hint file incorporated the amino acid sequences of leguminous plants using RefSeq data from the NCBI Reference Sequence Database for 6 species: *Senna tora*, common bean (*Phaseolus vulgaris*), peanut (*Arachis hypogaea*), chickpea (*Cicer arietinum*), soybean (*Glycine max*), and *Prosopis alba*. This hint file was generated using Exonerate ver.2.4.0^51^ and the ‘exonerate2hints.pl’ command in AUGUSTUS, enhancing the accuracy of gene prediction by integrating detailed protein sequence data into the draft genome annotations.

### 2.10. Transcriptome analysis

Preprocessed read data used for the Trinity assembly were mapped to the draft genome using Bowtie2 ver. 2.4.5.^52^ The output SAM file was sorted using SAMtools and converted into a BAM file. The resulting BAM files were used to count reads and to evaluate expression levels using StringTie ver. 2.2.1.^53^ The -e option command was employed to estimate the expression levels of each isoform in terms of FPKM and TPM. Further analysis was conducted using TPM values.

### 2.11. Orthologous gene prediction and phylogenetic analysis

Ortholog analysis was utilized to comprehensively predict the functions of genes in *C. siliqua* using SonicParanoid ver. 1.3.0.^54^ Ortholog groups were established based on the amino acid RefSeq data of *Physcomitrium patens*, *Marchantia polymorpha*, *Selaginella moellendorffii*, *Oryza sativa*, *Arabidopsis thaliana*, *S. tora*, *P. vulgaris*, *A. hypogaea*, *C. arietinum*, *G. max*, and *P. alba* and the predicted amino acid sequences of *C. siliqua*. Orthologous groups of genes associated with GM biosynthesis were identified using protein information from *A. thaliana* or *G. max*. Previously reported *C. tetragonoloba* ManS (accession no. CAI79402.1) and GMGT (accession no. AAR23313.1) sequences were integrated into each ortholog group for phylogenetic analysis on the NGPhylogeny.fr platform,^55^ and FastTree was used to generate phylogenetic trees using the maximum likelihood method.

### 2.12 Quantitative PCR

Total RNA (500 ng each) was used for cDNA synthesis using a PrimeScript RT Reagent kit (Takara, Otsu, Japan) following the manufacturer’s protocol. Reverse-transcription quantitative PCR (RT-qPCR) of each cDNA sample was performed using a Thermal Cycler Dice Real Time System III (Takara) using gene-specific primers and TB Green Fast qPCR Mix (Takara) and cycling conditions based on the manufacturer’s protocol. All primers were designed using Primer-BLAST^56^ based on the predicted gene sequences of *C. siliqua* (Supplementary Table S1); the predicted serine/threonine-protein phosphatase 4 like protein (*PP4L*) gene was used as an internal control. Results were analysed using the ΔΔCt method^57^ with software provided in the Thermal Cycler Dice Real Time System.

### 2.13 Quantitative analysis of GM

Quantitative analysis of GM was performed using 1 g of callus tissue and 0.005 g of endosperm tissue as described previously^58^ and analysed by LC-MS/MS. The calli and endosperm were boiled in 10 mL of water for 5 min, homogenized, and extracted with hot water for 20 min with stirring at 90°C. The supernatant was collected by centrifugation at 12,000 *g* for 10 min, and trichloroacetic acid was added to 10% (w/v) concentration. After placing the solution on ice for 30 min to precipitate the proteins, the supernatant was collected by centrifugation at 12,000 *g* for 10 min, and ethanol was added to 70% (v/v). The solution was incubated at −20 °C for 30 min and centrifuged at 12,000 *g* for 10 min. The supernatant was discarded, and the precipitate was washed with 70 % (v/v) ethanol and centrifuged at 12,000 g for 10 min, and the precipitate was collected. The precipitate was dissolved in 100 μL of 10 % (v/v) sulfuric acid and heated at 80 °C for 1 h to allow hydrolysis. Next, the solution volume was adjusted to 2 mL with distilled water, and the solution was filtered and used as sample. For the LBG standard, 4 mg of Locust Bean Gum A200 (Mitsubishi Chemical) was treated using the same method and adjusted to 20 mL with distilled water. Mannose and galactose standard samples were dissolved in ultrapure water and prepared at concentrations ranging from 0 to 100 µM each.

The samples were analysed using a Sciex 4000QTRAP hybrid triple quadrupole/linear ion trap mass spectrometer (AB Sciex, Framingham, MA, USA) under the conditions shown in Supplementary Table S2. Chromatographic separation was achieved using an ExionLC AE (AB Sciex) HPLC system equipped with an Ultron PS-80P HPLC column (300 × 8.0 mm, 10 μm) (Shinwa Chemical Industries, Kyoto, Japan) at 80 °C with a flow rate of 1.0 mL/min. The mobile phase was 100% ultrapure water, and the sample injection volume was 10 μL. Quantification was performed by multiple reaction monitoring (MRM) in negative mode to detect the specific precursor-to-product ion transitions *m*/*z* 179 → 89 for D-mannose and d-galactose. The compound-dependent parameters and source parameters for D-mannose and D-galactose are listed in Supplementary Table S2. The data were acquired using Analyst software ver. 1.5 (AB Sciex). Using mannose and galactose standards as external standards, calibration curves were created from the area under the curve, and the contents of these sugars in each sample were determined. Assuming that mannose originated from GM, the GM content was determined by adding the weight of one galactose to every 4 mannoses.

## 3. Results

### 3.1 Quantitative analysis of galactomannan

**Fig. 2.**
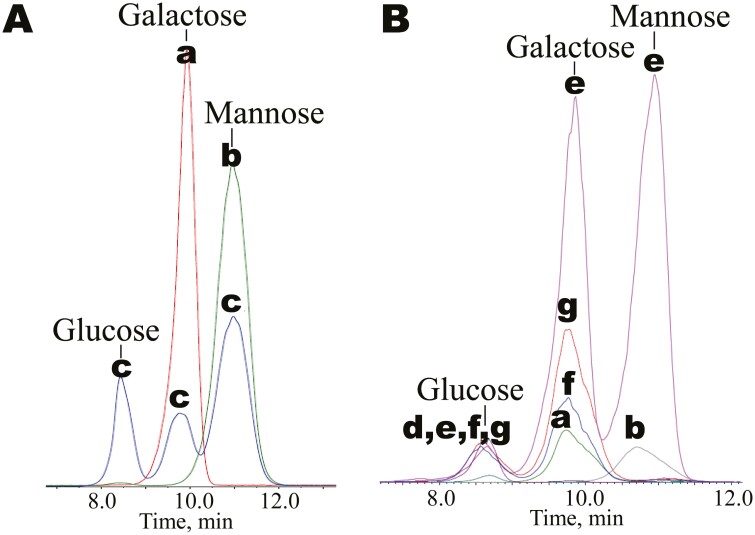
MRM chromatograms with the mass transition *m*/*z* 179/89 for aldohexose isomers. Lowercase letters indicate samples from which the peaks originate. (A) Overlay of chromatograms for the (a) galactose standard sample, (b) mannose standard sample, and (c) acid hydrolysate of LBG standard sample. (B) Overlay of chromatograms for the (a) galactose standard sample, (b) mannose standard sample, and acid hydrolysates of extracts from (d) stem, (e) endosperm, (f) leaf, and (g) callus tissues.

We detected glucose, galactose, and mannose in samples derived from the LBG standard. The ratio of galactose to mannose was approximately 1:4, which is consistent with previous findings (Table 1, Fig. 2). By contrast, we detected glucose and galactose in samples derived from *C. siliqua* leaves, stems, and calli, but mannose was not detected. On the other hand, glucose, galactose, and mannose were detected in samples derived from *C. siliqua* endosperm, with a galactose-to-mannose ratio of approximately 1:2. The GM content in the endosperm was 59 mg/g based on mannose content.

**Table 1.** Quantitative measurement of galactose and mannose derived from water-soluble polymers using LC-MS/MS. The content per unit wet weight of each tissue is indicated.

	Leaf (*n* = 3)		Stem (*n* = 3)		Callus (*n* = 3)		Endosperm (*n* = 3)	
	Mean	SD	Mean	SD	Mean	SD	Mean	SD
Galactose (μg/g)	0.77	0.05	0.15	0.12	51	0.52	3.8 × 10^4^	0.099 × 10^4^
Mannose (μg/g)	N/A		N/A		N/A		5.2 × 10^4^	0.20 × 10^4^

### 3.2 Genome assembly and gene prediction

Based on flow cytometry, the genome size of the sample was estimated to be 604 Mbp (Supplementary Fig. S2, Supplementary Table S3). The optimal *k*-mer size was estimated to be 119, and the genome assembly size was predicted to be 482 Mbp by analysing the short-read dataset sequence with KmerGenie (Supplementary Fig. S3A). The genome size estimated by GenomeScope was 459 Mbp, and heterozygosity was 0.345% (Supplementary Fig. S3B). To determine whether a program would be appropriate for assembling the sequencing dataset, we used publicly available assemblers (Supplementary Table S4). Assembly could be performed using ABySS with short-read sequences, whereas any assembler tested could be used to assemble a dataset of long-read sequences. The N50 value and longest contig length using Hifiasm was 36.0 and 60.6 Mbp, respectively; both values were higher than those obtained with the other assemblers (e.g. IPA generated an assembly with a contig N50 of 19.5 Mbp). The BUSCO score of the assembly was higher using Hifiasm (BUSCO score > 90 % for all 4 core gene sets) compared to IPA (BUSCO score = 78–86 %) (Supplementary Table S5). After removing reads derived from organelles, the final nuclear assembly obtained with Hifiasm was 496 Mbp long, which is close to the genome size estimated by KmerGenie, and consisted of 44 contigs (Table 2, Supplementary Fig. S4, and Supplementary Table S6). We predicted telomere regions at both ends of the 6 large contigs and on one side of the other 6 large contigs and 4 smaller contigs (Supplementary Table S6). The final genome assembly has BUSCO completeness scores of 98.4%, 97.1%, 96.7%, and 90.6% for the core gene sets of Eukaryota, Embryophyta, Eudicots, and Fabales, respectively (Table 2).

**Table 2.** Summary global statistics of the final draft genome assembly of *Ceratonia siliqua*.

Contig N50 length (Mbp)	36.0
Longest contig (Mbp)	60.6
Total length (Mbp)	496.0
Number of contigs	44
GC contents (%)	33.4
BUSCO statistic (Eukaryota_odb10)	98.4%
BUSCO statistic (Embryophyta_odb10)	97.1%
BUSCO statistic (Eudicots_odb10)	96.7%
BUSCO statistic (Fabales_odb10)	90.6%

To obtain the RNA assembly, we assembled the leaf-derived RNA sequence data using Trinity and generated 55,594 contigs with an average length of 856 bp (Supplementary Table S7). The Ex90N50 was 1.66 kbp, which is slightly longer than the N50, and a peak appeared around Ex93 (Supplementary Fig. S5).

To estimate the number of repetitive elements, we conducted a search for repeat elements and identified 2,273 *C. siliqua–*specific TE families generated *de novo*. The total interspersed repeat content accounted for 46.5% of the genome (Supplementary Table S8). We then predicted 35,796 gene models from the assembled genome sequence. Of these predicted genes, 32,730 showed similar amino acid sequences in the nr database, with a cut-off *E*-value of 1e-5, and 30,148 genes mapped to RNA-seq reads derived from leaf, stem, and callus tissue (Supplementary Fig. S6).

### 3.3 Functional annotation of proteins based on ortholog clusters

To annotate the predicted genes, we searched for orthologous genes from the assembled genome. A multi-species orthology inference using 660,197 proteins identified 16,908 ortholog groups (Table 3). Among these, 13,231 ortholog groups were found among *C. siliqua* proteins. All enzymes that function in the GM biosynthetic pathway were designated in *C. tetragonoloba*,^59^ but not all the corresponding genes have been reported. By searching for annotations of *A. thaliana* or *G. max* proteins using each enzyme name as a query, we identified ortholog groups containing enzymes associated with the GM biosynthesis pathway (Supplementary Table S9, Supplementary Fig. S7).^59^ These ortholog groups included the translated proteins of the *C. siliqua* assembled genome, indicating that the predicted genes were annotated to all enzymes listed in the GM pathway (Table 4).

**Table 3.** Statistics of comprehensive ortholog analysis using SonicParanoid.

Species	Proteome size	Input proteins	Orthologs	No. orthologs	Assigned (%)	^*^OGs with ortho from species	OGs with ortho from species (%)
***Physcomitrium patens***	17,773,595	35,000	25,599	9,401	73.14	8,647	51.14
***Marchantia polymorpha***	16,371,690	37,887	21,398	16,489	56.48	8,618	50.97
***Selaginella moellendorffii***	30,307,459	66,596	39,793	26,803	59.75	8,732	51.64
***Oryza sativa***	15,191,988	44,180	23,451	20,729	53.08	10,651	62.99
***Arabidopsis thaliana***	20,843,494	48,265	39,051	9,214	80.91	11,902	70.39
***Senna tora***	14,447,734	45,738	24,669	21,069	53.94	12,812	75.77
***Phaseolus vulgaris***	28,949,190	73,107	60,570	12,537	82.85	13,882	82.1
***Arachis hypogaea***	45,985,554	100,775	84,635	16,140	83.98	13,379	79.13
***Ceratonia siliqua***	17,842,853	41,072	31,284	9,788	76.17	13,231	78.25
***Cicer arietinum***	16,559,251	35,754	32,536	3,218	91	13,145	77.74
***Glycine max***	34,257,216	74,248	68,183	6,065	91.83	13,830	81.8
***Prosopis alba***	25,771,993	57,575	52,031	5,544	90.37	13,155	77.8
**Total**	2.84E + 08	660,197	503,200	156,997	76.22	16,908	100

^*^OGs, ortholog groups.

**Table 4.** Number of orthologs in ortholog groups encoding enzymes involved in the galactomannan biosynthesis pathway.

Enzymes related to GM biosynthesis	Number of orthologs		
	Ceratonia siliqua	Glycine max	Arabidopsis thaliana
Sucrose synthase	8	27	14
UDP-galactose 4-epimerase	4	8	6
Mannose-1-phosphate guanylyltransferase	1	4	4
Fructokinase	16	29	12
Glucokinase	6	14	10
Phosphomannomutase	3	6	5
Phosphomannose isomerase	2	3	4
Galactomannan galactosyltransferase	2	3	2
Aldose-1-epimerase	1	7	4
β-Galactosidase	5	6	10
α-Galactosidase	2	11	4
β-Mannosidase	12	25	6
Raffinose synthase	1	3	4
Galactokinase	2	2	1
Galactinol synthase	4	8	9
Stachyose synthase	1	1	2
Sucrose phosphate synthase	3	5	2
Sorbitol dehydrogenase	2	2	2
Sucrose phosphate phosphatase	4	12	16
Phosphoglucoisomerase	14	36	19
Galactose-1-phosphate uridylyltransferase	3	4	4
Invertase	5	17	9
Mannan synthase-like proteins (see below)			
Cellulose synthase-like A2 and A9	5	10	2
Cellulose synthase-like D2 and D3	2	8	2
Cellulose synthase-like D5	0	2	1
Glucomannan 4-beta-mannosyltransferase	1	2	0

**Fig. 3.**
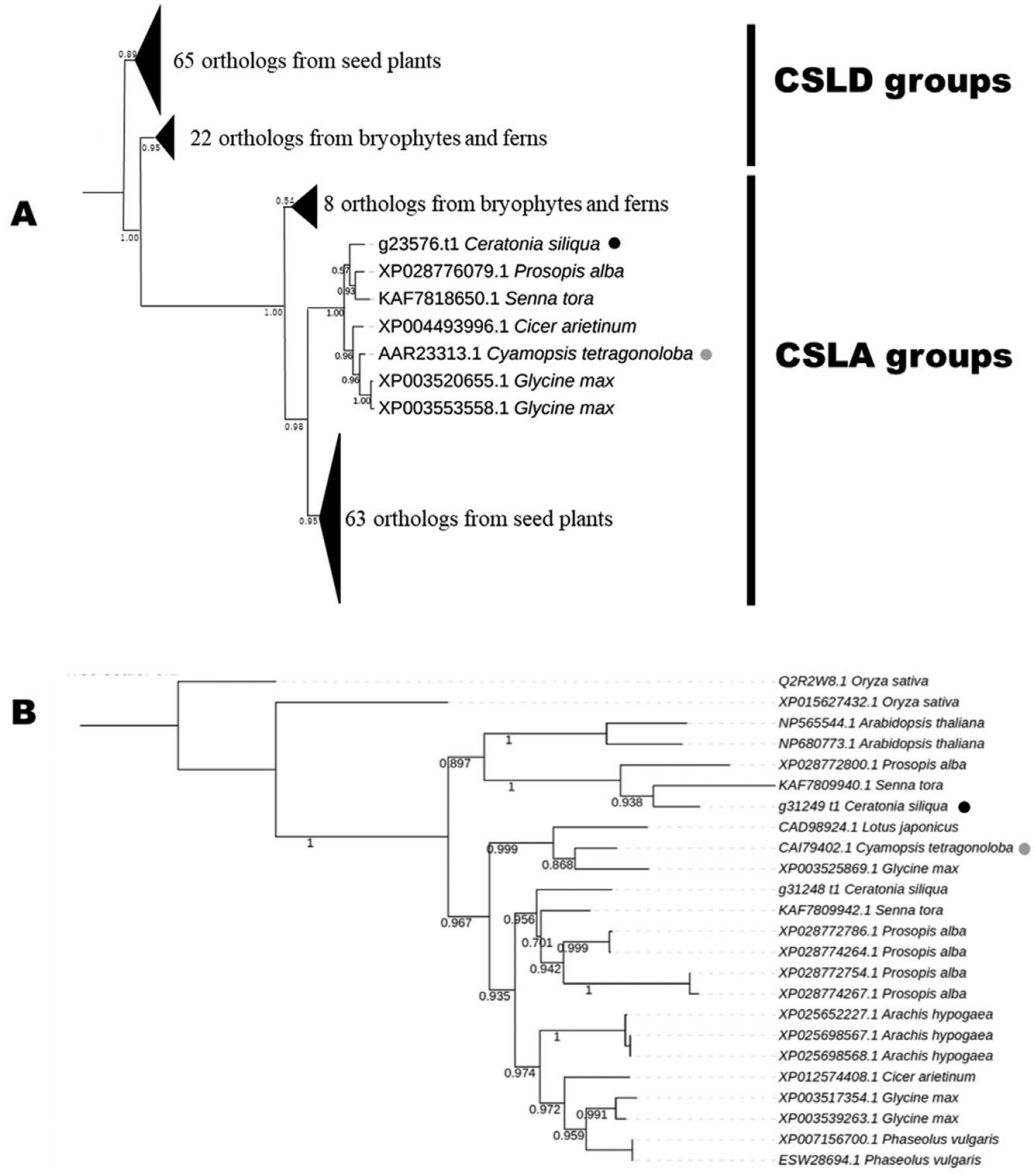
Phylogenetic trees of GMGT and ManS orthologs involved in galactomannan biosynthesis. (A) ML phylogenetic tree of the ManS ortholog group. The complete tree is shown in Supplementary Fig. S8. The grey circle indicates the ManS ortholog of *C. tetragonoloba*, and the black circle indicates the ManS ortholog of *C. siliqua*. (B) ML phylogenetic tree of the GMGT ortholog group. The grey circle indicates the GMGT ortholog of *C. tetragonoloba*, and the black circle indicates the GMGT ortholog of *C. siliqua*.

In the ortholog groups annotated as ManS and GMGT, there were 8 and 2 predicted *C. siliqua* genes, respectively (Table 4). A ManS (accession no. AAR23313.1) and a GMGT (accession no. CAI79402.1) exhibited enzymatic activity for synthesizing GM biochemically in *C. tetragonoloba*, suggesting that both proteins function in a final step for GM biosynthesis.^59^ To identify genes corresponding to these ManS and GMGT enzymes in *C. tetragonoloba*, we performed phylogenetic analysis of the genes in these ortholog groups (Supplementary Fig. S8). In the phylogenetic tree of ManS orthologs, one predicted gene (g23576.t1) formed a clade with the *C. tetragonoloba ManS* gene, with a high bootstrap value (Fig. 3A). Likewise, a GMGT clade formed with 2 neighbouring predicted genes (g31248.t1 and g31249.t1) separated into sister clades. g31248.t1 was placed closer to *C. tetragonoloba* GMGT, whereas neither appeared to be orthologous to *C. tetragonoloba* GMGT based on their relationships to *G. max* genes and genes from species outside Fabaceae (Fig. 3B).

### 3.4 Transcriptome analysis and RT-qPCR of genes associated with GM biosynthesis

To identify genes associated with GM biosynthesis, we analysed gene expression profiles using RNA-seq read counts. Genes with higher expression levels in the endosperm than in other tissues encoded sucrose synthase, mannose-6-phosphate isomerase, β-galactosidase, aldose-1-epimerase, β-mannosidase, stachyose synthase, galactinol synthase, UDP-galactose 4-epimerase, and phosphoglucoisomerase (Supplementary Fig. S9). Conversely, genes with lower expression levels in the endosperm than in other tissues encoded glucokinase, sorbitol dehydrogenase, invertase, sucrose phosphate phosphatase, galactose-1-phosphate uridylyltransferase, and fructokinase (Supplementary Fig. S10). Genes with no significant differences in transcript levels between endosperm and other tissues encoded phosphomannomutase, mannose-1-phosphate guanylyltransferase, sucrose phosphate synthase, galactokinase, raffinose synthase, and α-galactosidase (Supplementary Fig. S11).

**Fig. 4.**
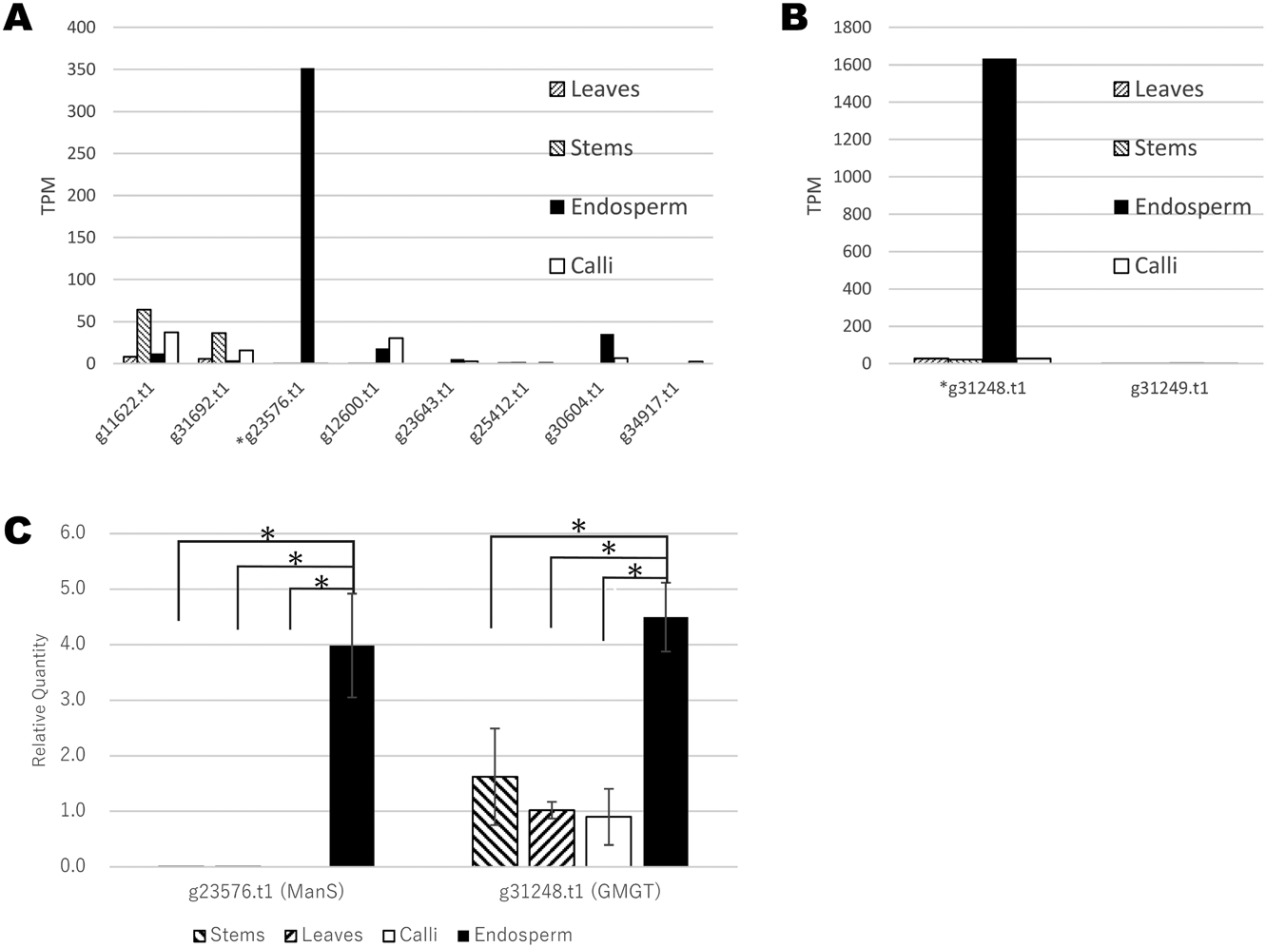
The transcript levels of orthologous genes classified as ManS and GMGT genes. (A) RNA-seq of ManS orthologs. (B) RNA-seq of GMGT orthologs. Gene expression levels are shown as normalized TPM values. (C) RT-qPCR of transcript levels of the predicted *ManS* gene (g23576.t1) and the predicted *GMGT* gene (g31248.t1). Total RNA was extracted from stems, leaves, calli, and endosperm and subjected to RT-qPCR. The transcript levels were normalized to *PP4L* transcript levels. Error bars represent SD of 3 biological repeats (*P < 0.01).

We examined the transcript levels of predicted genes classified in the ManS and GMGT ortholog groups (Fig. 4A, B). Of these predicted genes, both a ManS gene (g23576.t1) and a GMGT gene (g31248.t1), which clustered with *C. tetragonoloba* genes, were highly expressed specifically in endosperm. The other GMGT gene (g31249.t1) was not highly expressed. In agreement with the RNA-seq data, RT-qPCR revealed that transcripts of these ManS and GMGT orthologs were more abundant in endosperm compared to other tissues (Fig. 4C).

## 4. Discussion

### 4.1 GM accumulation in endosperm

We performed simplified quantification of GM by decomposing this molecule into monosaccharides and measuring mannose, which constitutes the major backbone chain. Mannose was detected only in extracted water-soluble polymers from endosperm containing mannose. In contrast, GM was undetectable in extracts from leaves, stems, or calli (Fig. 2), although mannose itself was detected in the supernatant during ethanol precipitation, confirming its presence in these tissues as a monosaccharide (data not shown). These results suggest that GM predominantly accumulates in endosperm but not in leaves, stems, or calli, which is consistent with the notion that GM functions as a storage polysaccharide in endosperm.^3^ The composition ratio of galactose to mannose derived from water-soluble polymers in the endosperm (1:2) was higher than that of the LBG standard (1:4), perhaps because galactose in the soluble polymer fraction was derived not only from GM but also from other polysaccharides such as pectin, and the LBG standard was prepared in a different manner from the galactose and mannose standards.

In the total ion chromatogram (Supplementary Fig. S12), the detection of numerous ions originating from impurities around the peaks of galactose and mannose highlighted the effectiveness of the MRM measurements. The use of MRM makes quantification possible even with a relatively simple sample pre-treatment method. This newly established method allows the presence of GM to be predicted based on the detection of mannose. More specific quantification using degradation analysis at the oligosaccharide level via enzymatic hydrolysis could be performed in the future.

### 4.2 Draft nuclear genome assembly

The previously reported genome size of *C. siliqua* varies depending on the habitat, ranging from 583 to 614 Mbp,^60^ which is consistent with our estimated size (604 Mbp) based on flow cytometry (Supplementary Table S3). Only one peak was detected in the counts and was assumed to be 2C (Supplementary Fig. S2). The predicted genome assembly size (482 Mbp) based on sequencing reads was smaller than the genome size estimated by flow cytometry.

Hifiasm and IPA, which are capable of separating haplotypes, produced an assembly with exceptionally good contiguity (Supplementary Table S4). Because maternal and paternal genetic information was unavailable for the locust bean samples used in this study, a ‘primary assembly’ was used for the following analyses. Due to the high completeness scores obtained using BUSCO, we generated a draft nuclear genome for *C. siliqua* that was assembled by Hifiasm. Of the 44 assembled contigs, 13 contigs longer than 15 Mbp (the maximum length is 60.6 Mbp, contig N50 size of 36.0 Mbp) were identified. Of these 13 contigs, 6 contained putative telomere regions at both ends, and 6 contained these regions at one end. These results suggest that the *de novo* genome assembly represents 6 chromosomal sequences and that the others are still fragmented, possibly due to the presence of ribosomal RNA repeats or centromeric regions. An initial attempt to construct a pseudochromosome-level draft genome using chromosome interaction mapping (Hi-C)^61^ with a conventional sequence library construction method failed, likely due to the interference of abundant impurities derived from the plant tissues. The telomere search revealed that 22 telomere regions in the contigs. Because the haploid chromosome number of *C. siliqua* is 12,^62^ this is two fewer than expected. All detected telomere regions contained the repeat sequence TTTAGGG (CCCTAAA), which is common in angiosperms. Additionally, the size of telomeric regions was approximately 16 kbp, which is similar to sequence read length. One possibility is that some subtelomic regions were very similar such that the reads covering the missing telomere regions were assembled into the same contig. For example, if the telomere is connected with the rRNA repeats, it would be difficult to resolve. A much less likely possibility might be that a new type of terminal structure is present. Some hint might be obtained with cytogenetics studies involving *in situ* hybridization and ultra-long read sequencing.

The contigs assembled with the HiFi reads presented in this study exhibited significantly greater contiguity than the publicly available Illumina WGS assembly (accession no. GCA_034509205.1)^17^ with a scaffold N50 of 18.8 kbp (Supplementary Table S10). Furthermore, our contigs scored ~8% higher in the BUSCO completeness assessments than the Illumina scaffolds (Table2 vs Supplementary Table S10), suggesting higher suitability as the basis to construct genomic information resources and perform molecular analyses.

In RNA-seq *de novo* assembly, a graph displaying the ExN50 metric (which incorporates expression levels) indicates that the quality of the assembly improves as the peak approaches Ex90, provided that the sequence depth is adequate.^46^ The clear peak near Ex90 in the RNA *de novo* assembly of *C. siliqua* and the BUSCO score of > 80% suggest that a high-quality assembly was generated. The repeat element content of *C. siliqua* is similar to that of the closely related species *S. tora*,^63^ which also belongs to the Caesalpinioideae. We also used these results as a hint file for gene prediction with AUGUSTUS, resulting in 35,796 predicted genes. Transcriptome reads were mapped to these 30,148 genes, suggesting that the prediction results are reliable.

### 4.3 Predicted genes associated with the GM biosynthesis pathway

We predicted orthologous groups of identified genes that might be associated with the GM biosynthesis pathway in *C. siliqua*, as inferred from gene annotations in other model plants (Table 4). Additionally, we identified a clade consisting of the genes encoding ManS, including a predicted gene (g23576.t1) from *C. siliqua*, along with genes from *G. max*, *C. tetragonoloba*, *S. tora*, *P. alba*, and *C. arietinum*, using phylogenetic analysis. These leguminous plants produce GM in their seeds.^64–66^ Transcriptome profiling and RT-qPCR indicated that the predicted *C. siliqua* gene is predominantly expressed in endosperm. Therefore, the genes in this clade may play roles in mannan synthesis in endosperm, but this notion awaits experimental confirmation. The predicted gene (g31248.t1) from *C. siliqua* forms a clade of GMGT orthologs, including a gene (gmgt1; CAI79402) from *C. tetragonoloba* whose draft genome was recently reported.^67^ This *C. tetragonoloba* gene is expressed in developing seeds.^68^ Similarly, we detected the accumulation of transcripts of the predicted gene in *C. siliqua* endosperm. The genes in this group are not exact orthologs of gmgt1, but it appears that this gene family has expanded in leguminous plants that produces GM in their seeds. Perhaps this gene family has expanded to fulfil the strong need for GM biosynthesis in endosperm.

To further explore the GM biosynthesis mechanism in *C. siliqua*, it will be important to conduct functional analysis of the genes presented here. One approach for the functional analysis of these genes is to demonstrate the ability of the encoded proteins to synthesize GM *in vitro*. Another approach is to assess whether these genes are involved in GM biosynthesis *in vivo* using transgenic or mutagenic approaches. However, this will be challenging because transgenic techniques for *C. siliqua* are not currently available, and *C. siliqua* has a long life cycle. Nonetheless, such analyses will shed light on the industrial application of these genes for GM production.

The draft genome and transcriptome data produced in this study could facilitate the development of transgenic methods and transgene-free genome editing via native promoter identification and guide RNA design. Finally, endosperm-specific gene expression data could help elucidate why *C. siliqua* produces high levels of high-quality LBG that could be used for industrial purposes, unlike the GMs of other leguminous plants. It might be possible in the future to produce GM more efficiently in endosperm-like cultured cells or genetically modified legumes.

## Supplementary Material

dsae033_suppl_Supplementary_Material

## Data Availability

The raw sequencing reads have been deposited in DDBJ/ENA/GenBank under accession number DRA015129. The assembly data have been deposited in DDBJ/ENA/GenBank under accession number BTHC01000001-BTHC01000044.
