# Supplementary material for: De novo sequencing allows genome-wide identification of genes involved in galactomannan synthesis in locust bean (Ceratonia siliqua)
Source: DNA Res. 2024 Dec 2;31(6):dsae033. doi: 10.1093/dnares/dsae033 (PMC11659883; doi:10.1093/dnares/dsae033)
Supplement: dsae033_suppl_Supplementary_Material [file dsae033_suppl_supplementary_material.docx]

Supplementary Materials for

***De novo* sequencing allows genome-wide identification of genes involved in galactomannan synthesis in locust bean (*Ceratonia siliqua*)**

**Table of Contents**

**Supplementary tables**

[Supplementary Table S1. The primers used for qRT-PCR. 3](#_Toc183621377)

[Supplementary Table S2. Compound dependent parameters for D-mannose and IS in MRM mode for LC–MS/MS analysis. 4](#_Toc183621378)

[Supplementary Table S3. Mean fluorescent intensity and corresponding genome size of reference plants, and estimated genome size of *C. siliqua* by flow cytometry. 5](#_Toc183621379)

[Supplementary Table S4. Summary statistics for the draft genome assembly of *C. siliqua*. 6](#_Toc183621380)

[Supplementary Table S5. BUSCO statistics for assembly by Hifiasm and IPA. 7](#_Toc183621381)

[Supplementary Table S6. List of contigs and telomere analysis results. 8](#_Toc183621382)

[Supplementary Table S7. Stats of RNA assembly obtained by Trinity. 9](#_Toc183621383)

[Supplementary Table S8. Statistics of repetitive elements. 10](#_Toc183621384)

[Supplementary Table S9. The number of orthologs in orthologous groups containing the enzyme annotated to be involved in galactomannan biosynthesis. 11](#_Toc183621385)

[Supplementary Table S10. Statistics of the Illumina *C. siliqua* whole genome shotgun assembly. 12](#_Toc183621386)

[Supplementary Fig. S1. Assembly and analysis pipeline. 13](#_Toc183621387)

[Supplementary Fig. S2. Flow cytometry of PI-stained nuclei from *C. siliqua.* 14](#_Toc183621388)

[Supplementary Fig. S3. Best k-mer prediction and genome size estimation. 15](#_Toc183621389)

[Supplementary Fig. S4. The schematics of draft genome obtained by Hifiasm. The graphs were drawn using Bandage. 16](#_Toc183621390)

[Supplementary Fig. S5. ExN50 plots generated by Trinity. 17](#_Toc183621391)

[Supplementary Fig. S6. Venn diagram of transcriptome analysis. 18](#_Toc183621392)

[Supplementary Fig. S7. The galactomannan biosynthesis pathway of *C. tetragonoloba*. 19](#_Toc183621393)

[Supplementary Fig. S8. Phylogenetic tree of the ManS orthologous group. 20](#_Toc183621394)

[Supplementary Fig. S9. The transcript levels of orthologous genes in orthologous groups containing the enzyme annotated to be involved in galactomannan biosynthesis pathway. 21](#_Toc183621395)

[Supplementary Fig. S10. The transcript levels of orthologous genes in orthologous groups containing the enzyme annotated to be involved in galactomannan biosynthesis pathway. 22](#_Toc183621396)

[Supplementary Fig. S11. The transcript levels of orthologous genes in orthologous groups containing the enzyme annotated to be involved in galactomannan biosynthesis pathway. 23](#_Toc183621397)

[Supplementary Fig. S12. The total ion chromatogram (TIC) and mass spectrum from the endosperm. 24](#_Toc183621398)

[Reference 25](#_Toc183621399)

# **Supplementary Table S1.** The primers used for qRT-PCR.

| **Genes** | **Primer sequences** | | **Sizes** |
| --- | --- | --- | --- |
|  | **Forward** | **Reverse** | **(bp)** |
| **PP4L** | **5’-AAGCAGGTCGTGGAGTTGAG** | **5’-ACCCTTCCTCCCAACCAAAC** | **96** |
| **ManS** | **5’-ACCAGCCAGCGTGATAGTTC** | **5’-GTAGATGCAGGGACCTTGGG** | **113** |
| **GMGT** | **5’-TTGGGAGGAGATTGTGGGGA** | **5’-ACTAACCTTCTCTGCGTGCC** | **103** |

Supplementary Table S2. Compound dependent parameters for D-mannose and IS in MRM mode for LC–MS/MS analysis. The analysis parameters for galactose and mannose were optimized to identical numerical values across all instances.

| **Compound** | **D-mannose / D-galactose** |
| --- | --- |
| [M-H]- | 178.8 |
| MRM transition | 89.0 |
| Ion spray voltage | -4500 V |
| Ion source temperature | 600 °C |
| Ion source gas1 | 40 |
| Ion source gas1 | 30 |
| Curtain gas | 10 |
| Collision gas | 4 |
| Declustering Potential | -55 |
| Entrance Potential | -10 |
| Collision Energy | -12 |
| Collision Cell Exit Potential | -13 |

Supplementary Table S3. Mean fluorescent intensity and corresponding genome size of reference plants, and estimated genome size of *C. siliqua* by flow cytometry.

| **Species** | **Genom size** | **Mean fluorescence intensity (2C DNA)** | **Coefficient of variation** |
| --- | --- | --- | --- |
| *Oryza sativa* | 389 Mbp | 277338 | 4.82% |
| *Arabidopsis thaliana* | 135 Mbp | 104672 | 6.36% |
| *Brachypodium distachyon* | 272 Mbp | 225393 | 6.80% |
| *Solanum lycopersicum* | 950 Mbp | 616652 | 3.69% |
| *Glycine max* | 1120 Mbp | 778391 | 4.43% |
| *Ceratonia siliqua* | 604 Mbp | 420625 | 3.83% |

Supplementary Table S4. Summary statistics for the draft genome assembly of *C. siliqua*. The assembly by Hifiasm is shown in gray.

|  | Assembler | Contig N50 length (Mbp) | Longest contig (Mbp) | Total length (Mbp) | Number of contigs |
| --- | --- | --- | --- | --- | --- |
| Long-read  assembler | Raven | 0.11 | 0.89 | 550 | 6070 |
|  | Flye | 0.65 | 13.3 | 611 | 9125 |
|  | Canu | 0.08 | 6.5 | 940 | 17063 |
|  | Mecat2 | 5.39 | 17.1 | 483 | 871 |
|  | Wtdbg2 | 1.11 | 11.1 | 612.79 | 2614 |
|  | Apollo | 0.11 | 0.89 | 547 | 6070 |
|  | Rust-mdbg | 0.01 | 0.04 | 1218 | 3772433 |
|  | JumboDB | 0.01 | 0.05 | 58290 | 4392900 |
|  | IPA | 17.5 | 59.3 | 493 | 506 |
|  | Hifiasm | 36 | 60.6 | 511 | 460 |
| Short-read  assembler | Abyss | 0.02 | 0.4 | 598 | 297844 |
|  | Velvet | out of memory error | |  |  |
|  | Soap2denovo | out of memory error | |  |  |

Supplementary Table S5. BUSCO statistics for assembly by Hifiasm and IPA.

|  |  | | Completeness | | |
| --- | --- | --- | --- | --- | --- |
| **Core gene set** | Core genes | Hifiasm | | | IPA |
| **Eukaryota_odb10** | 255 | 98.4% | | | 85.5％ |
| **Embryophyta_odb10** | 1614 | 97.3% | | | 81.7％ |
| **Eudicots_odb10** | 2326 | 96.7% | | | 82.9％ |
| **Fabales_odb10** | 5366 | 90.2% | | | 77.6％ |
|  |  | |  |  |  |

# **Supplementary Table S6.** List of contigs and telomere analysis results. * "One" indicates that the telomere region was detected on only one end. ** "Both" indicates that the telomere regions were detected at both ends.

| Contig ID | Length  (Mbp) | Telomere detection | Contig ID | Length  (Mbp) | Telomere detection |
| --- | --- | --- | --- | --- | --- |
| ptg000012 | 60.6 | *One | ptg000019 | 0.301 | - |
| ptg000004 | 60.1 | *Both | ptg000038 | 0.228 | - |
| ptg000008 | 43.4 | One | ptg000032 | 0.138 | - |
| ptg000003 | 40.0 | Both | ptg000036 | 0.124 | - |
| ptg000014 | 36.3 | Both | ptg000035 | 0.101 | - |
| ptg000016 | 36.0 | Both | ptg000031 | 0.098 | - |
| ptg000017 | 35.4 | Both | ptg000033 | 0.096 | - |
| ptg000007 | 34.8 | Both | ptg000039 | 0.068 | - |
| ptg000023 | 29.9 | One | ptg000040 | 0.056 | - |
| ptg000025 | 29.1 | One | ptg000009 | 0.051 | - |
| ptg000002 | 28.5 | One | ptg000022 | 0.047 | - |
| ptg000011 | 18.9 | One | ptg000027 | 0.042 | - |
| ptg000028 | 16.5 | - | ptg000024 | 0.040 | - |
| ptg000005 | 9.3 | - | ptg000021 | 0.036 | - |
| ptg000001 | 5.1 | One | ptg000013 | 0.034 | - |
| ptg000006 | 3.9 | - | ptg000043 | 0.028 | - |
| ptg000010 | 2.6 | One | ptg000041 | 0.027 | - |
| ptg000018 | 1.9 | - | ptg000030 | 0.023 | - |
| ptg000034 | 0.8 | - | ptg000020 | 0.021 | - |
| ptg000029 | 0.6 | One | ptg000044 | 0.021 | - |
| ptg000015 | 0.5 | One | ptg000042 | 0.020 | - |
| ptg000026 | 0.3 | - | ptg000037 | 0.011 | - |

Supplementary Table S7. Stats of RNA assembly obtained by Trinity.

| Average (kbp) | 0.86 |
| --- | --- |
| Contig N50 length (kbp) | 1.55 |
| Longest contig (kbp) | 13.6 |
| Total length (kbp) | 47,610.16 |
| GC contents (%) | 41.21 |
| Number of contigs | 55,594 |
| Ex90N50 (kbp) | 1.66 |
| Ex90 number of genes | 12,212 |
| BUSCO statistic (Eukaryota_odb10) | 98.0% |
| BUSCO statistic (Embryophyta_odb10) | 88.7% |
| BUSCO statistic (Eudicots_odb10) | 86.8% |
| BUSCO statistic (Fabales_odb10) | 80.4% |

Supplementary Table S8. Statistics of repetitive elements.

|  | Number of elements | Total(bp) | % of genome |
| --- | --- | --- | --- |
| SINEs | 833 | 87,417 | 0.02 |
| LINEs | 5,736 | 2,125,012 | 0.43 |
| LTR | 81,932 | 88,446,326 | 17.83 |
| DNA elements | 25,640 | 14,849,279 | 2.99 |
| Unclassified | 459,251 | 125,161,954 | 25.23 |
| total interspersed repeats | - | 230,669,988 | 46.50 |
| Small RNA | 1,414 | 3,015,017 | 0.61 |
| Satellites | 0 | 0 | 0 |
| Simple repeats | 120,896 | 5,500,936 | 1.11 |
| Low complexity | 26,614 | 1,373,002 | 0.28 |

Supplementary Table S9. The number of orthologs in orthologous groups containing the enzyme annotated to be involved in galactomannan biosynthesis.

| GM synthesis related protein |  | Number of orthologs | | | | | | | | | | | |
| --- | --- | --- | --- | --- | --- | --- | --- | --- | --- | --- | --- | --- | --- |
|  | ***Arachis hypogaea*** | | ***Ceratonia siliqua*** | ***Cicer arietinum*** | ***Glycine max*** | ***Senna tora*** | ***Prosopis alba*** | ***Phaseolus vulgaris*** | ***Arabidopsis thaliana*** | ***Oryza sativa*** | ***Selaginella moellendorffii*** | ***Marchantia polymorpha*** | ***Physcomitrium patens*** |
| Sucrose synthase | 13 | | 8 | 8 | 27 | 5 | 14 | 20 | 14 | 11 | 3 | 1 | 7 |
| UDP-galactose 4-epimerase | 10 | | 4 | 3 | 8 | 5 | 10 | 10 | 6 | 4 | 4 | 4 | 7 |
| Mannose-1-phosphate guanylyltransferase | 8 | | 1 | 2 | 4 | 1 | 5 | 4 | 4 | 1 | 3 | 2 | 3 |
| Fructokinase | 25 | | 16 | 13 | 29 | 6 | 13 | 28 | 12 | 8 | 9 | 4 | 14 |
| Glucokinase | 20 | | 6 | 6 | 14 | 4 | 17 | 16 | 10 | 14 | 5 | 4 | 7 |
| Phosphomannomutase | 8 | | 3 | 2 | 6 | 3 | 10 | 6 | 5 | 2 | 7 | 4 | 5 |
| Phosphomannose isomerase | 2 | | 2 | 1 | 3 | 2 | 3 | 2 | 4 | 1 | 3 | 2 | 2 |
| Galactomannan galactosyltransferase | 3 | | 2 | 1 | 3 | 2 | 5 | 2 | 2 | 2 | 0 | 0 | 0 |
| Aldose-1-epimerase | 2 | | 1 | 5 | 7 | 1 | 3 | 4 | 4 | 0 | 0 | 0 | 0 |
| β-Galactosidase | 13 | | 5 | 5 | 6 | 5 | 4 | 15 | 10 | 3 | 10 | 4 | 4 |
| α-Galactosidase | 6 | | 2 | 4 | 11 | 2 | 12 | 5 | 4 | 5 | 6 | 0 | 3 |
| β-Mannosidase | 15 | | 11 | 6 | 25 | 11 | 11 | 17 | 6 | 3 | 6 | 4 | 7 |
| Raffinose synthase | 2 | | 1 | 4 | 3 | 1 | 4 | 2 | 4 | 1 | 0 | 0 | 0 |
| Galactokinase | 4 | | 2 | 1 | 2 | 2 | 5 | 2 | 1 | 1 | 4 | 4 | 2 |
| Galactinolsynthase | 9 | | 4 | 3 | 8 | 3 | 6 | 6 | 9 | 2 | 6 | 1 | 0 |
| Stachyose synthase | 4 | | 1 | 1 | 1 | 1 | 2 | 4 | 2 | 0 | 0 | 0 | 0 |
| Sucrose phosphate synthase | 4 | | 3 | 3 | 5 | 2 | 5 | 4 | 2 | 2 | 2 | 2 | 4 |
| Sorbitol dehydrogenase | 7 | | 2 | 1 | 2 | 1 | 11 | 2 | 2 | 2 | 2 | 1 | 2 |
| Sucrose phosphate phosphatase | 8 | | 4 | 8 | 12 | 2 | 19 | 6 | 16 | 2 | 3 | 2 | 5 |
| Phosphoglucoisomerase | 26 | | 14 | 15 | 36 | 10 | 20 | 24 | 19 | 6 | 7 | 0 | 11 |
| Galactose-1-phosphate uridylyltransferase | 4 | | 3 | 4 | 4 | 2 | 2 | 4 | 4 | 2 | 7 | 3 | 3 |
| Invertase | 12 | | 5 | 2 | 17 | 1 | 11 | 4 | 9 | 2 | 4 | 2 | 6 |
| Mannan synthase-like proteins (see below) |  | |  |  |  |  |  |  |  |  |  |  |  |
| Cellulose synthase like A2 and A9 | 10 | | 5 | 5 | 11 | 5 | 14 | 8 | 2 | 3 | 4 | 1 | 3 |
| Cellulose synthase like D2 and D3 | 21 | | 2 | 6 | 8 | 1 | 6 | 8 | 2 | 1 | 6 | 8 | 8 |
| Cellulose synthase like D5 | 2 | | 0 | 1 | 2 | 1 | 2 | 2 | 1 | 1 | 0 | 0 | 0 |
| Glucomannan 4-beta-mannosyltransferase | 0 | | 1 | 1 | 2 | 1 | 1 | 0 | 0 | 0 | 0 | 0 | 0 |

Supplementary Table S10. Statistics of the Illumina *C. siliqua* whole genome shotgun assembly (accession no. GCA_034509205.1).

| Genome size | 477.3 Mb |
| --- | --- |
| Total ungapped length | 471.8 Mb |
| Number of scaffolds | 99,288 |
| Scaffold N50 | 18.8 kb |
| Scaffold L50 | 3,416 |
| GC contents (%) | 33.0 |
| BUSCO statistic (Eukaryota_odb10) | 90.1% |
| BUSCO statistic (Embryophyta_odb10) | 84.7% |
| BUSCO statistic (Eudicots_odb10) | 86.5% |
| BUSCO statistic (Fabales_odb10) | 83.4% |


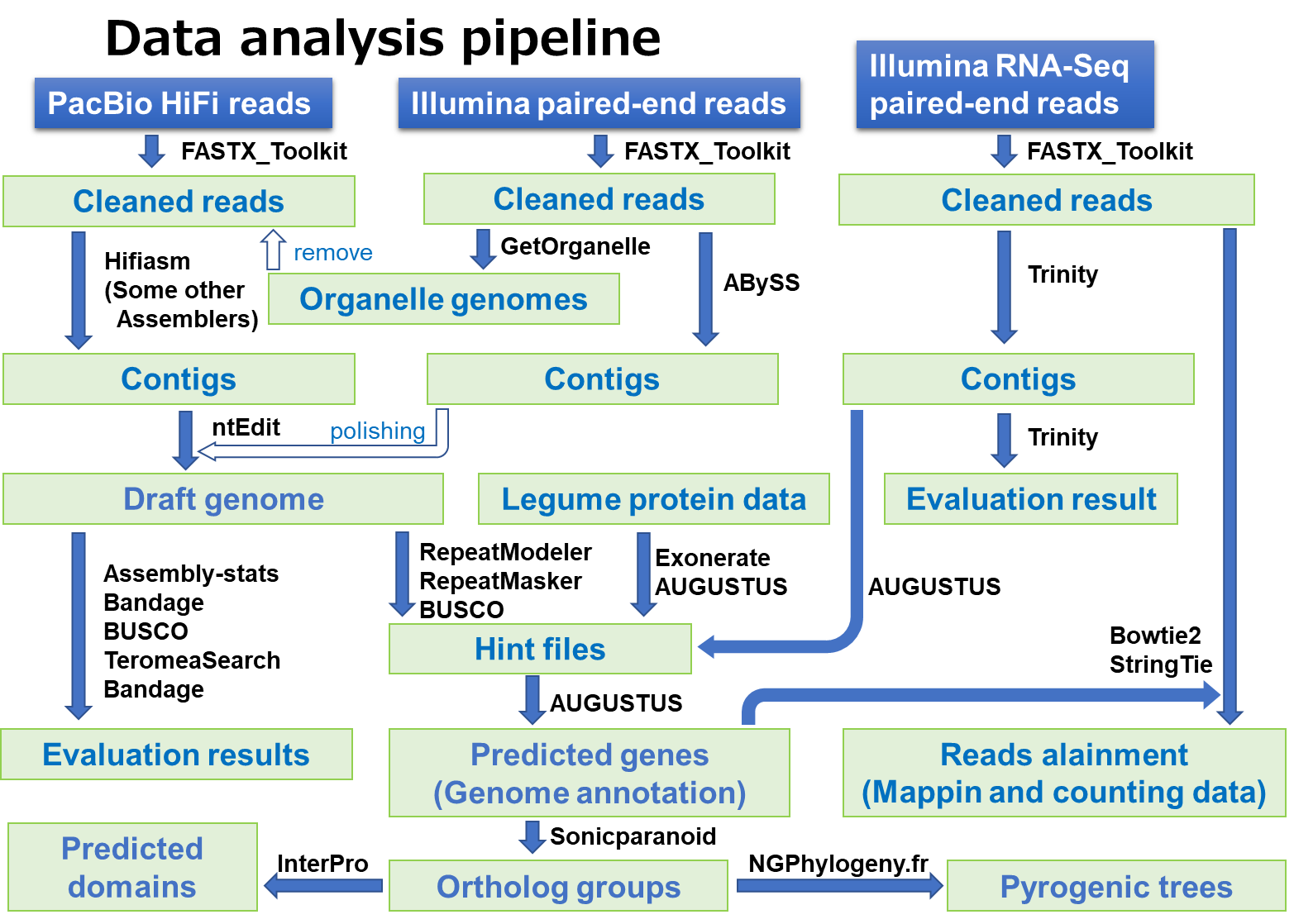


Supplementary Fig. S1. Assembly and analysis pipeline.


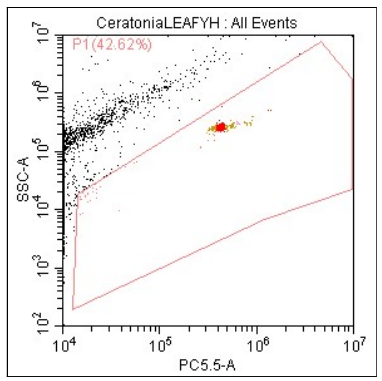

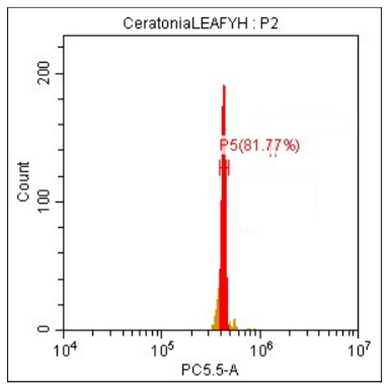


Supplementary Fig. S2. Flow cytometry of PI-stained nuclei from *C. siliqua.*

**
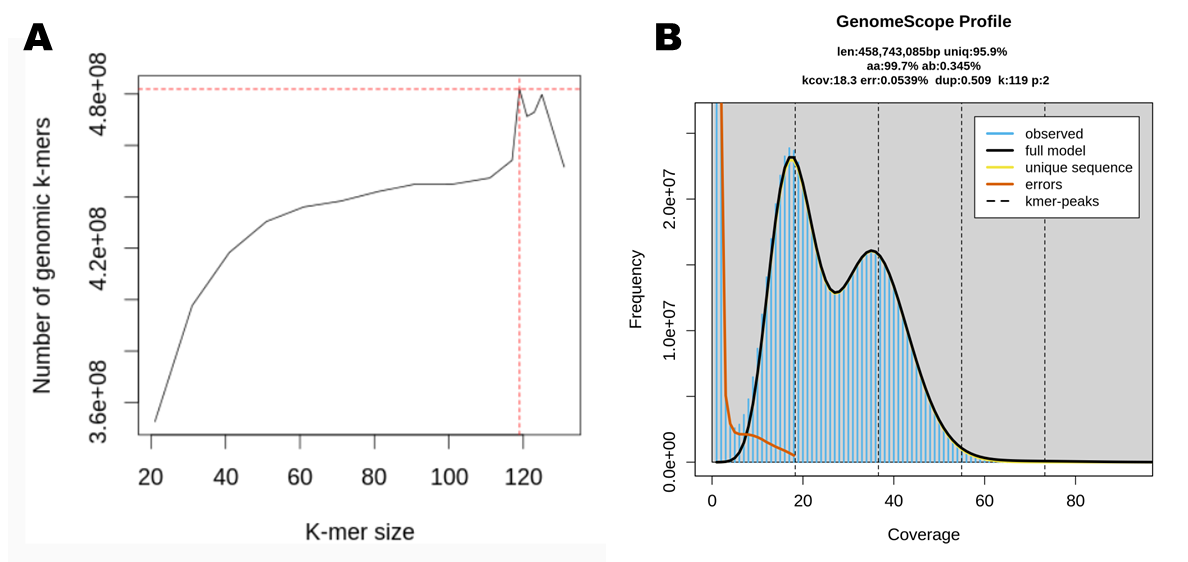
**

Supplementary Fig. S3. Best k-mer prediction and genome size estimation.

(A) KmerGenie analysis results. Predicted best K-mer size was 119, and predicted assembly size was 481.9 Mbp.

(B) GenomeScope analysis results. Estimated genome size was 459 Mbp. In the diagram, abbreviations represent the following parameters: 'len' – inferred total genome length; 'uniq' – percentage of the genome that is unique (non-repetitive); 'aa' – overall homozygosity rate; 'ab' – overall heterozygosity rate; 'kcov' – mean k-mer coverage for heterozygous bases; 'err' – read error rate; 'dup' – average rate of read duplications; 'k' – k-mer size; 'p' – ploidy.


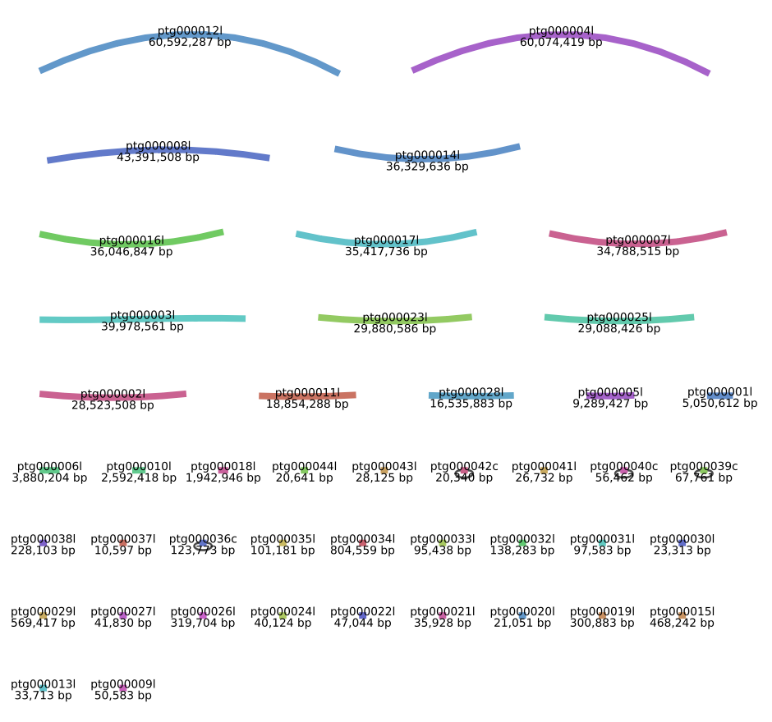


Supplementary Fig. S4. The schematics of draft genome obtained by Hifiasm. The graphs were drawn using Bandage.

Supplementary Fig. S5. ExN50 plots generated by Trinity.

**
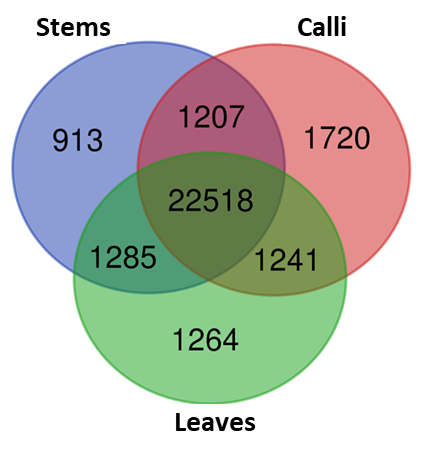
**

Supplementary Fig. S6. Venn diagram of transcriptome analysis. Venn diagram representing the number of predicted genes that RNA-seq reads were mapped. The RNA-seq reads derived from leaves, stems and calli were mapped respectively.

**
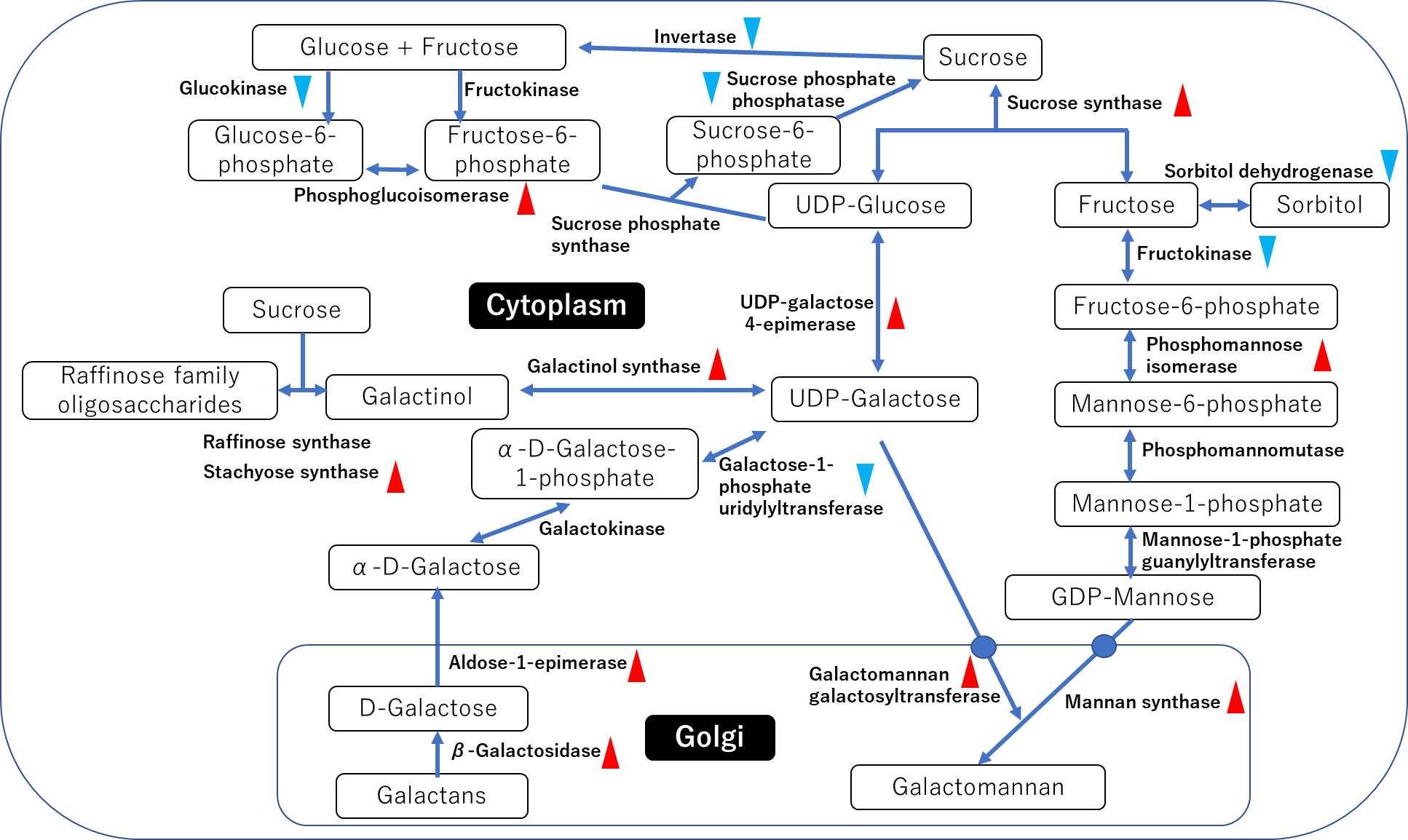
**

Supplementary Fig. S7. The galactomannan biosynthesis pathway of *C. tetragonoloba* ^1^. The triangles indicate enzyme containing orthologs with high transcription levels in the endosperm of *C. siliqua* in RNA-Seq analysis. The inverted triangles indicate enzyme containing orthologs with low transcription levels in the endosperm of *C. siliqua* in RNA-Seq analysis.

**
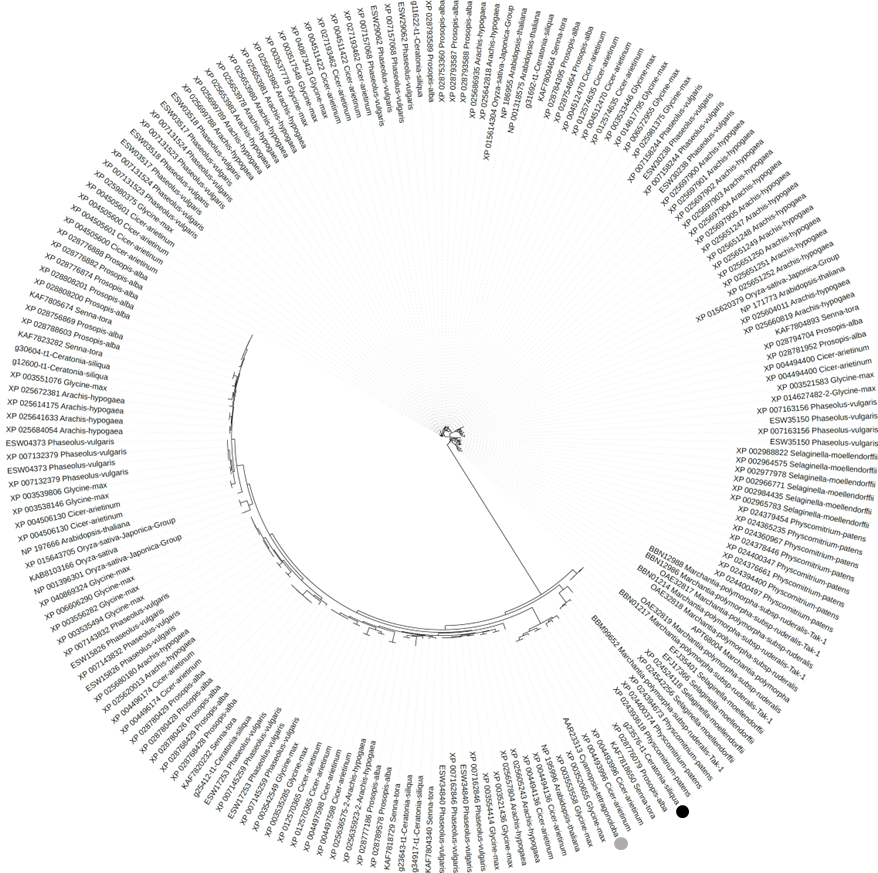
**

# **Supplementary Fig. S8.** Phylogenetic tree of the ManS orthologous group. The gray circle indicates the ManS ortholog of *C. tetragonoloba* and the black circle indicates *C. siliqua* ManS ortholog. ta of *P. patens, M. polymorpha, S. moellendorffii, O. sativa, A. thaliana, S. tora, P. vulgaris, A. hypogaea, C. arietinum, G. max, P. alba, C. tetragonoloba* and *C. siliqua* are included as taxon.

**
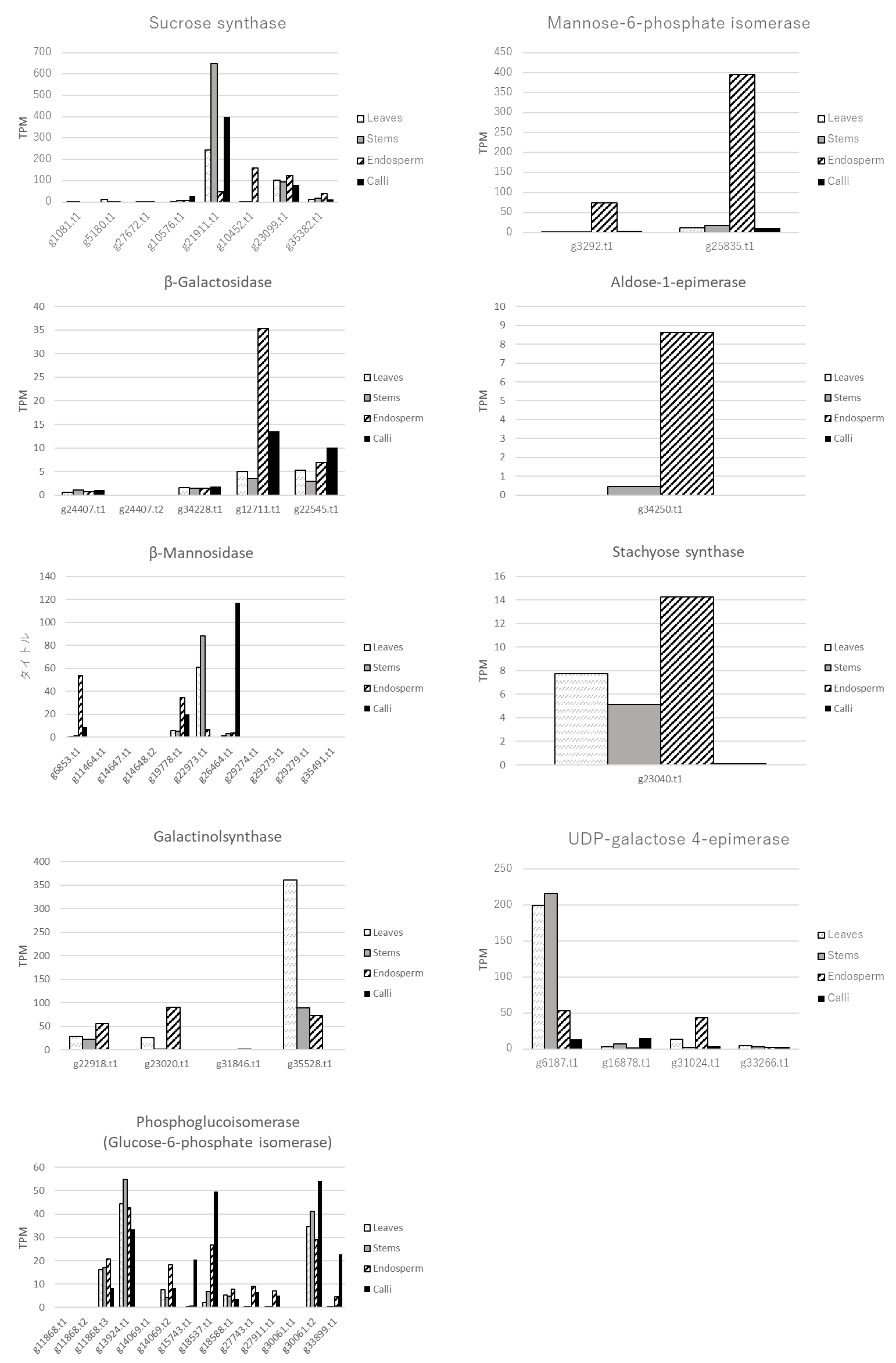
**

# **Supplementary Fig. S9.** The transcript levels of orthologous genes in orthologous groups containing the enzyme annotated to be involved in galactomannan biosynthesis pathway. The orthologous groups containing orthologous genes with high transcription levels only in the endosperm are shown.

**
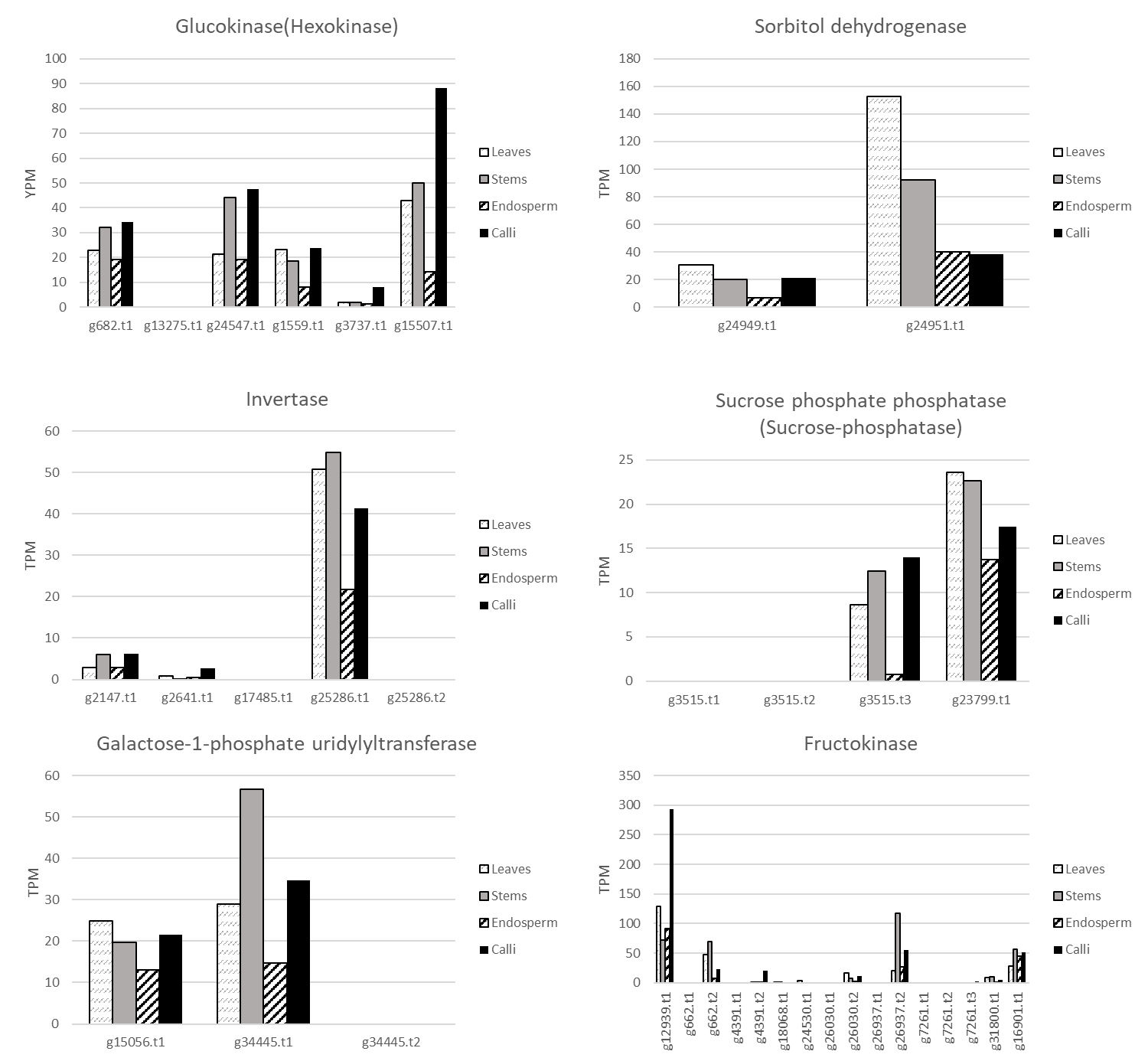
**

# **Supplementary Fig. S10.** The transcript levels of orthologous genes in orthologous groups containing the enzyme annotated to be involved in galactomannan biosynthesis pathway. The orthologous groups containing orthologous genes with low transcription levels only in the endosperm are shown.

**
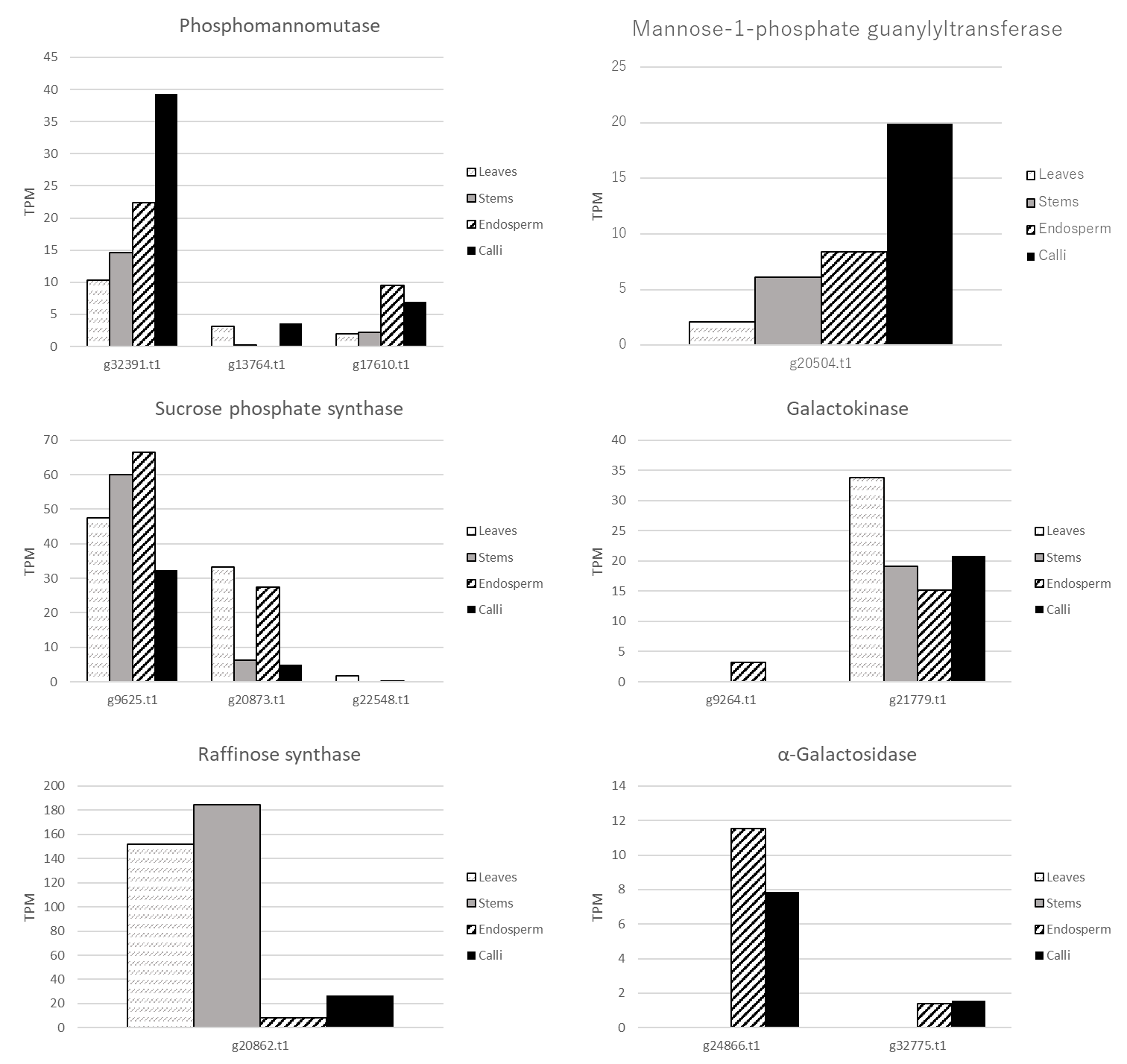
**

Supplementary Fig. S11. The transcript levels of orthologous genes in orthologous groups containing the enzyme annotated to be involved in galactomannan biosynthesis pathway. The orthologous groups containing orthologous genes with no significant differences in transcript levels between endosperm and other tissues are shown.

**
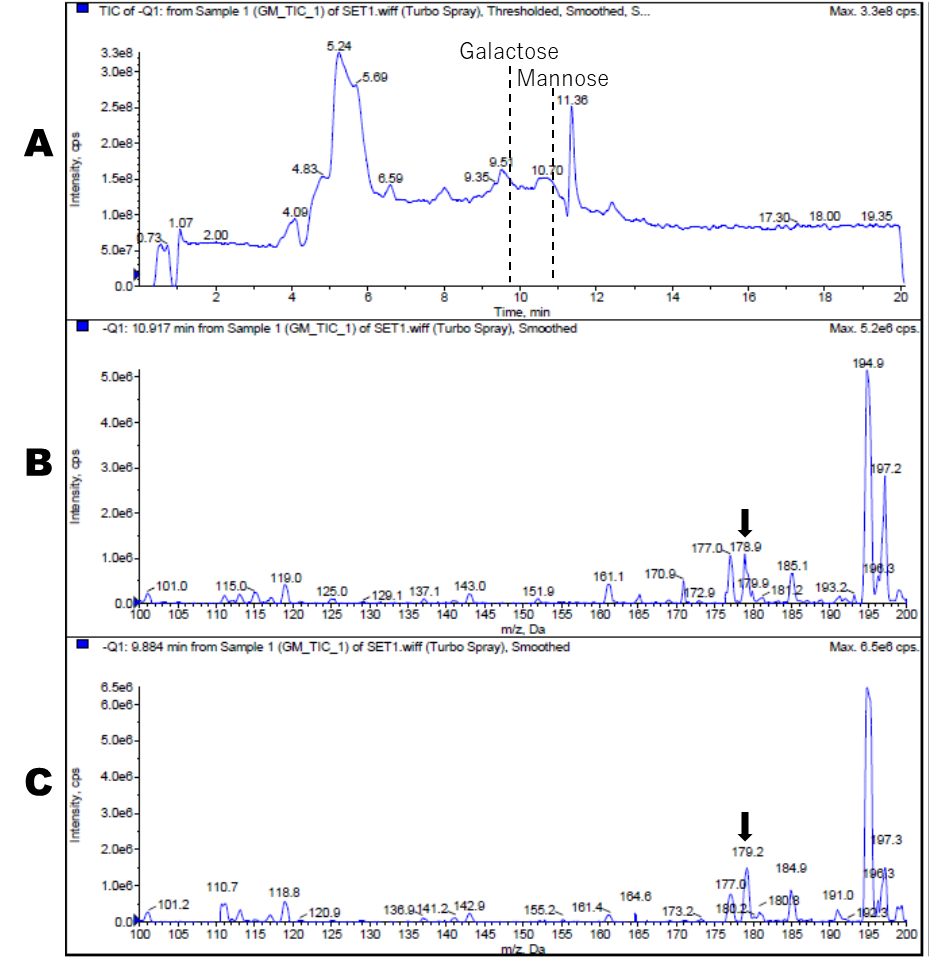
**

Supplementary Fig. S12. The total ion chromatogram (TIC) and mass spectrum from the endosperm.

(A) The TIC of endosperm extract hydrolysates. The dotted lines indicate the retention times of mannose and galactose, respectively. (B) Mass spectrum corresponding to the retention time observed for mannose. The black arrow denotes the position at 178.8 m/z, corresponding to the ion derived from mannose. (C) Mass spectrum corresponding to the retention time observed for galactose. The black arrow denotes the position at 178.8 m/z, corresponding to the ion derived from galactose.

Despite the ion detection range being narrowed to 100-200 m/z, the TIC exhibited a high baseline, which obscured the peaks at the retention times associated with mannose and galactose. Additionally, numerous ions, presumably derived from impurities, were observed in the mass spectra.

Reference

1. Sharma, P., Sharma, S., Ramakrishna, G., Srivastava, H. and Gaikwad, K. 2022, A comprehensive review on leguminous galactomannans: structural analysis, functional properties, biosynthesis process and industrial applications. *Crit. Rev. Food Sci. Nutr.*, **62**, 443-465.
